# Induction of endoplasmic reticulum stress and mitochondrial dysfunction dependent apoptosis signaling pathway in human renal cancer cells by norcantharidin

**DOI:** 10.18632/oncotarget.23465

**Published:** 2017-12-19

**Authors:** Min-Hua Wu, Hui-Ling Chiou, Chu-Liang Lin, Ching-Yi Lin, Shun-Fa Yang, Yi-Hsien Hsieh

**Affiliations:** ^1^ Institute of Medicine, Chung Shan Medical University, Taichung, Taiwan; ^2^ Department of Laboratory, Chung-Kang Branch, Cheng-Ching General Hospital, Taichung, Taiwan; ^3^ School of Medical Laboratory and Biotechnology, Chung Shan Medical University, Taichung, Taiwan; ^4^ Institute of Biochemistry, Microbiology and Immunology, Chung Shan Medical University, Taichung, Taiwan; ^5^ Division Of Chest Medicine, Department of Internal Medicine, Taichung Veterans General Hospital, Taichung, Taiwan; ^6^ Department of Medical Research, Chung Shan Medical University Hospital, Taichung, Taiwan; ^7^ Department of Biochemistry, School of Medicine, Chung Shan Medical University, Taichung, Taiwan; ^8^ Clinical Laboratory, Chung Shan Medical University Hospital, Taichung, Taiwan

**Keywords:** norcantharidin, apoptosis, renal cancer cells, mitochondrial depolarization, endoplasmic reticulum

## Abstract

Previous studies reported that norcantharidin (NCTD) has anti-tumor effects. We investigated the antitumor effects and underlying mechanism of NCTD on human renal cancer *in vitro* and *in vivo*. NCTD significantly decreased renal cancer cell viability by induction of apoptosis, as determined by the MTT assay and annexin V/PI staining. NCTD treatment of 786-O and A-498 cells altered the expression of caspase family proteins and PARP. Moreover, NCTD induced mitochondrial depolarization, which was accompanied by an increased level of Bax and decreased levels of Bcl-2 and Mcl-1. NCTD induced endoplasmic reticulum (ER) stress by increasing the expression of Grp78, p-elF2α, ATF4, and CHOP. Pretreatment with an ER stress inhibitor (salubrinal) significantly attenuated the effect of NCTD. NCTD also induced activation of the AKT pathway in 786-O and A-498 cells. Overexpression of AKT partly reversed the effect of NCTD on apoptosis. NCTD treatment led to decreased expression of Bcl-2 and Mcl-1, and increased expression of Bax, cleaved-caspase-9, cleaved-PARP, and p-elF2α. Our *in vivo* studies demonstrated that NCTD significantly inhibited tumor growth in a nude mouse xenograft model. Taken together, our results suggest that NCTD is a potential anti-tumor agent for treatment of renal carcinoma.

## INTRODUCTION

Renal cell carcinoma (RCC) is the ten most frequently occurring human cancers [1]. RCC develops from the proximal renal tubular epithelial cells of the kidneys, and accounts for about 85% of renal cancers [2]. The current treatments consist of chemotherapy and immunotherapy, and are associated with numerous toxicities [3]. Therefore, it is necessary to better understand the molecular mechanisms of this cancer so that new anti-RCC molecular targets can be identified and effective and less toxic drugs can be developed.

**Figure 1:**
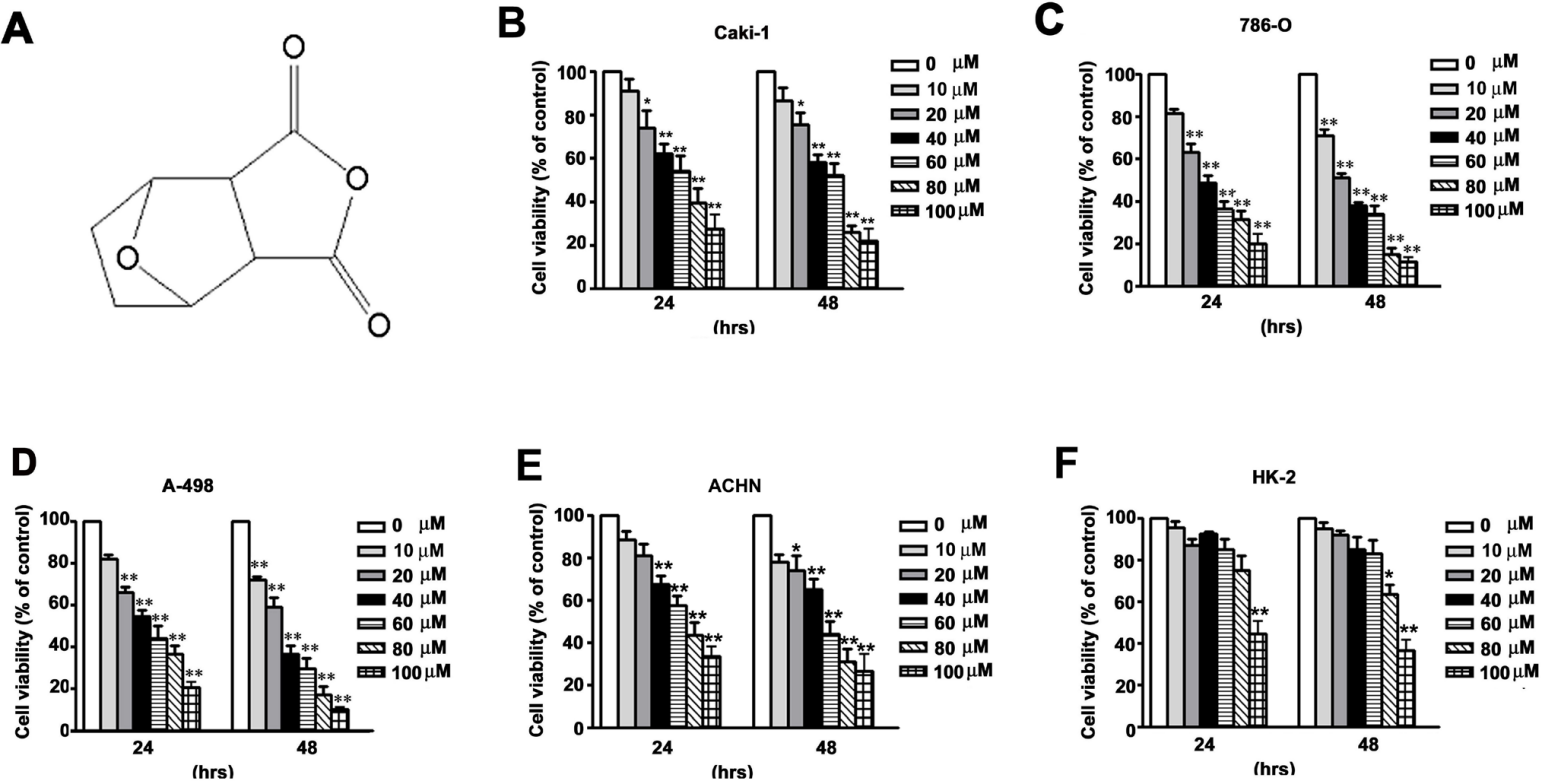
NCTD inhibits cell viability in human renal cancer cells (**A**) The chemistry structure of NCTD. (**B**–**E**) Four renal cancer cells (786-O, CaKi-1, ACHN and A-498) and (**F**) Normal immortalized proximal tubule epithelial cells (HK-2) were incubated with different concentration of NCTD for 24 and 48 h. Cell viability was measured using a MTT assay. All data are represented as mean ± SEM (*n* = 3) for each group. ^**^p < 0.01 compared with control.

Norcantharidin (NCTD, Figure 1A), the demethylated analog of cantharidin, is isolated from natural blister beetles [4]. NCTD has anti-tumor [5], pro-apoptotic [6, 7], anti-metastatic [8], and anti-angiogenic [9] effects. NCTD also has diverse anticancer activities against various types of tumor cells, such as prostate cancer, lung cancer, breast cancer, and colorectal carcinoma [10–13]. NCTD induces apoptosis through regulation of cell cycle-related proteins, leading to cell cycle arrest at the G2/M phase [12, 14]. Other studies reported that NCTD induces mitochondria-dependent apoptosis through inhibition of the AKT/FOXO4/Mcl-1 signaling pathway in human prostate cancer [15] and induces production of reactive oxygen species (ROS) via upregulation of the p38 MAPK pathway in human urinary bladder carcinoma cells [16]. Furthermore, a combination treatment consisting of NCTD with ABT-737 has increased therapeutic efficacy against HCC cells [17]. Nevertheless, the underlying molecular mechanisms of the anti-tumor effects of NCTD on renal cancer cells are still unknown.

Previous studies demonstrated that an imbalance of proteins in the Bcl-2 family, which have roles in the intrinsic apoptosis pathway, leads to increased mitochondrial permeability, release of cytochrome c, and increased caspase-9/caspase-3 expression [18]. There is also evidence that induction of endoplasmic reticulum (ER) stress proteins has a role in apoptosis in various cancer cells [19, 20]. The UPR involves three proteins, including inositol-requiring enzyme-1 (IRE1), activating transcription factor 6 (ATF6) and PKR-like ER kinase (PERK)-respond to the accumulation of unfolded proteins as part of a survival response [21]. When the intracellular misfolded or unfolded protein accumulation, ER releases a large number of Bip protein to help the accumulation of protein folding. The accumulation of unfolded protein is reduced, the ER function is restored [22]. So far, no studies have yet examined the effect of NCTD on induction of ER-stress induced apoptosis of tumor cells. The present study examines the anticancer activity of NCTD against renal cancer cells *in vitro* and *in vivo*.

## RESULTS

### NCTD reduces viability of human renal cancer cells

We initially investigated the effect of NCTD on the viability of four human renal cancer cells (786-O, A-498, CaKi-1, and ACHN) and normal proximal tubule epithelial cells (HK-2) using the MTT assay. NCTD reduced the viability of each of the four cell lines of renal cancer cells in a time- and concentration-dependent manner (Figure 1B–1E). However, the same concentrations and treatment durations only had a mild cytotoxic effect in the normal proximal tubule epithelial (HK-2) cells (Figure 1F). The IC50 values in the renal cancer cells and proximal tubule epithelial cells were 62.4 ± 5.7 μM (CaKi-1), 43.8 ± 6.8 μM (786-O), 48.2 ± 3.1 μM (A-498), 67.2 ± 3.8 μM (ACHN), and 96.5 ± 2.4 μM (HK-2) after 24 h treatment; 51.2 ± 4.1 μM (CaKi-1), 31.5 ±4.5 μM (786-O), 32.1 ± 4.2 μM (A-498), 56.2 ± 5.1 μM (ACHN), and 88.3 ± 3.8 μM (HK-2) after 48 h treatment, respectively (Figure 1B–1F). These data suggest that NCTD selectively inhibits renal cancer cells at concentrations that are not toxic to normal proximal tubule epithelial (HK-2) cells.

### NCTD induces apoptosis and alters expression of apoptosis-associated proteins in human renal cancer cells

**Figure 2:**
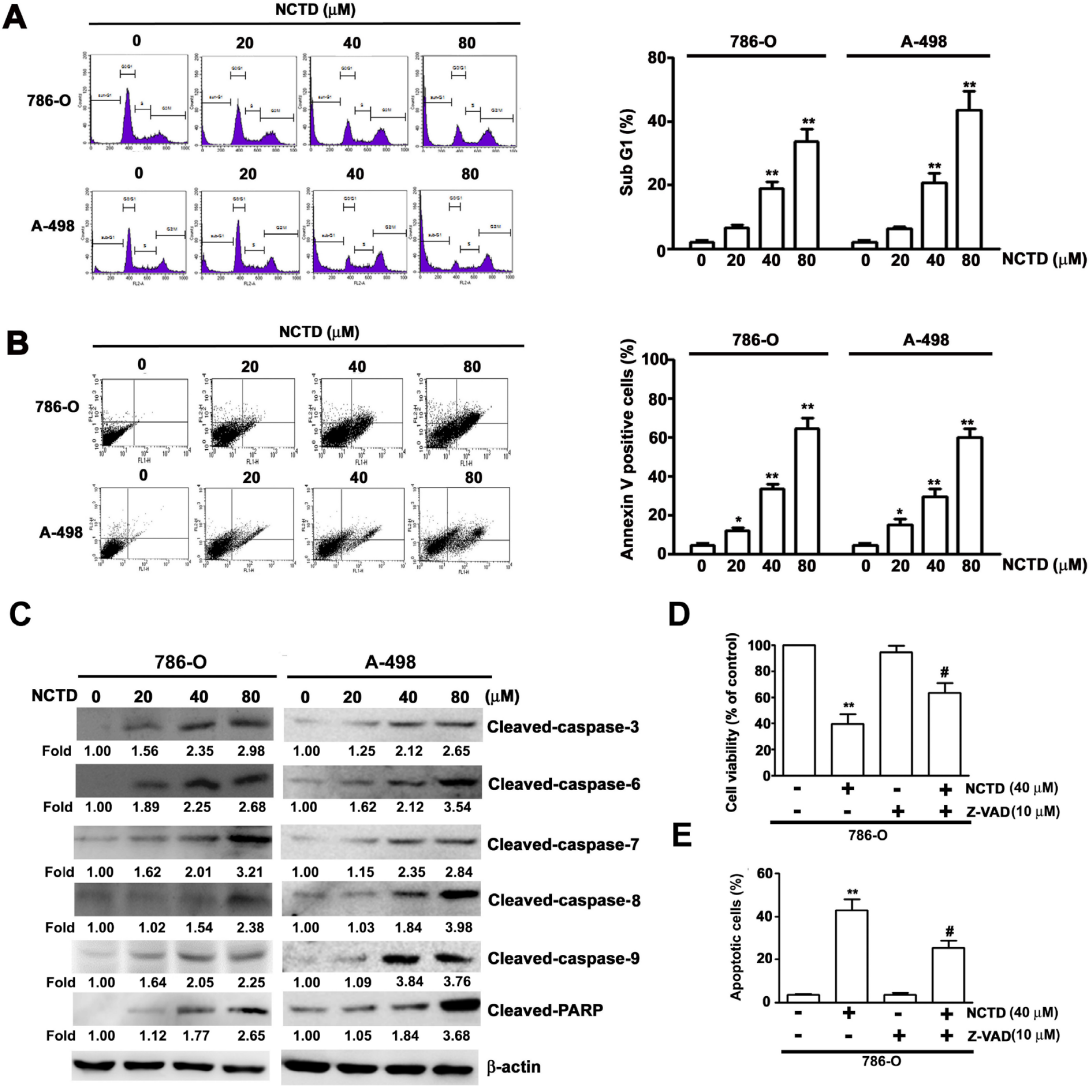
NCTD induces cell cycle arrest and apoptosis in 786-O and A-498 cells (**A**) 786-O and A-498 cells were incubated with NCTD (0, 20, 40 and 80 μM) for 24 h, then cell cycle distribution was performed by flow cytometry. (**B**) Induction of cell apoptosis of 786-O and A-498 cells were measured with Annexin-V and PI double-stained flow cytometry after treated with NCTD. (**C**) 786-O and A-498 cells were treated NCTD for 24 h to detect the expressions of caspases and PARP were detected by western blot analysis. β-actin used as an internal control. (**D**) Pretreated with pan-caspase (Z-VAD-FMK) for 2 h, then treated with NCTD (40 μM) for another 22 h. Cell viability was measured by the MTT assay. (**E**) Apoptotic cells were detected by the Annexin-V and PI double-stained flow cytometry. All data are represented as mean ± SEM (*n* = 3) for each group.^**^p < 0.01 compared with control. ^#^*p* < 0.01 compared with NCTD.

We next determined the effect of NCTD on induction of apoptosis in 786-O and A-498 cells by use of flow cytometry with propidium iodide (PI) staining (Figure 2A). The results indicate that NCTD led to an accumulation of cells in sub-G1 phase after 24 h in a concentration-dependent manner. In particular, the percentage of 786-O cells at sub-G1 was 20.3% at 40 μM NCTD and 36.8% at 80 μM NCTD; the percentage of A-498 cells at sub-G1 was 21.5% at 40 μM NCTD and 43.5% at 80 μM NCTD. We also examined the effect of NCTD on 786-O and A-498 using Annexin V/PI staining (an apoptosis assay) with flow cytometry. The results indicate that the number of Annexin V-FITC and PI positive cells increased as NCTD concentration increased (Figure 2B). We examined the effect of NCTD on the expression of several critical apoptosis-related proteins (caspase-3, caspase-6, caspase-7, caspase-8, caspase-9, and PARP) in 786-O and A-489 cells. NCTD induced accumulation of cleaved PARP and of activated caspases in a concentration-dependent manner (Figure 2C). To confirm the contribution of caspase activation on NCTD-induced apoptosis, we pretreated 786-O cells with a pan-caspase inhibitor (Z-VAD-FMK) markedly reversed the effect of NCTD on cell viability (Figure 2D) and apoptosis (Figure 2E). These *in vitro* results confirm that NCTD induces apoptosis in renal cancer cells.

**Figure 3:**
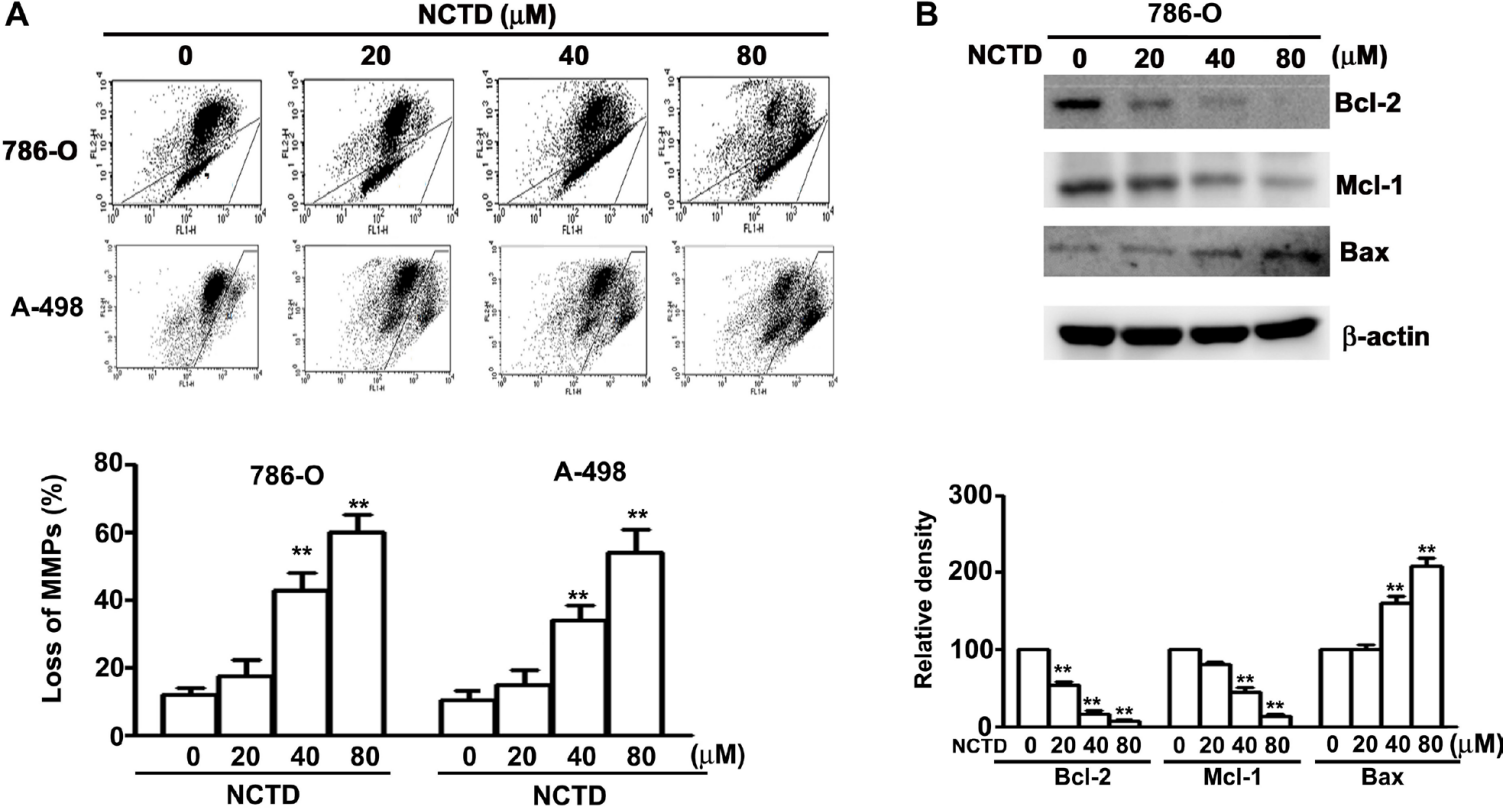
NCTD induces mitochondria-dependent apoptosis in 786-O and A-498 cells (**A**) Cells were incubated with NCTD (0, 20, 40 and 80 μM) for 24 h and stained with JC-1 reagent by flow cytometry. (**B**) Western blotting analysis of the expression levels of Bcl-2, Mcl-1 and Bax protein. All data are represented as mean ± SEM (*n* = 3) for each group. ^**^p < 0.01 compared with control. ^#^*p* < 0.01 compared with NCTD.

The loss of mitochondrial membrane potential can be triggered by the imbalance of Bax/Bcl-2 leading to the activated process of caspase-9. As shown in Figure 3A, NCTD induced a dose-dependent reduction in mitochondrial membrane potential in human renal cancer cells. We also found that NCTD upregulated Bax expression and downregulated Bcl-2 and Mcl-1 expression in a concentration-dependent manner (Figure 3B). These *in vitro* results demonstrate that NCTD-induced apoptosis accompanies mitochondrial dysfunction in human renal cancer cells.

### NCTD induces endoplasmic reticulum stress in 786-O cells

**Figure 4:**
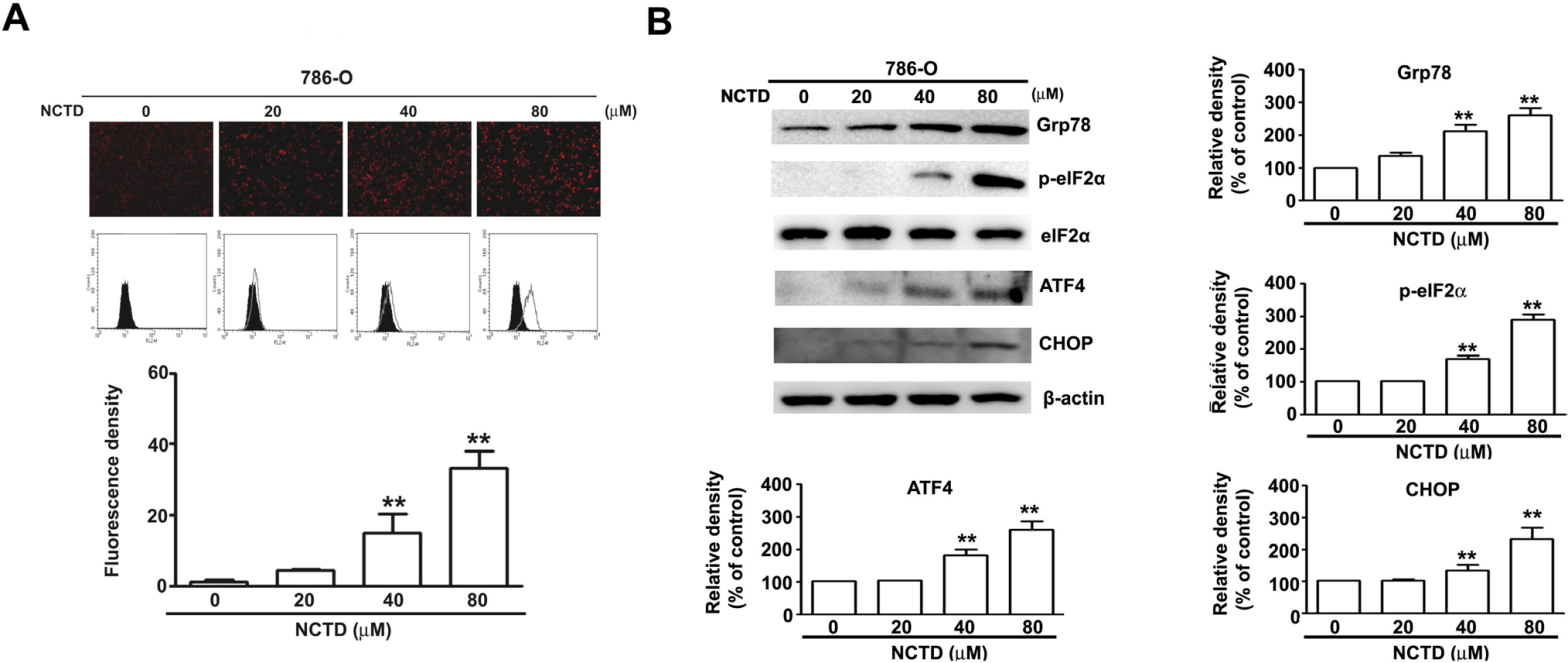
NCTD induce endoplasm reticulum stress in 786-O cells (**A**) 786-O cells were incubated with NCTD (0, 20, 40 and 80 μM) for 24 h. (**B**) The protein expression level of Grp78, ATF-4, CHOP, p-eIF2α and eIF2α were assessed by western blotting, β-actin used as an internal control. All data are represented as mean ± SEM (*n* = 3) for each group. ^**^p < 0.01 compared with control.

Several studies have reported the induction of ER stress during the apoptosis of various tumor cells [23]. Thus we determined the effect of NCTD on ER stress using GFP- labeled endoplasmic reticulum as an intracellular probe to assess stress level. The results show that GFP fluorescence increased in a concentration-dependent manner after NCTD treatment for 24 h, indicative of increased ER stress (Figure 4A). Several previous studies have previously reported that an unfolded protein response (UPR) induces PERK-mediated phosphorylation of eukaryotic initiation factor-2α, and the preferential translation of ATF-4 [23]. Grp78 is required to restore ER function, and ATF-4 also induces the expression of the transcriptional regulator CHOP, leading to induction of apoptosis [24]. Our results indicate that NCTD significantly increased the expression of Grp78, p-eIF2α, ATF-4, and CHOP in a concentration-dependent manner in 786-O cells (Figure 4B). These *in vitro* results suggest that NCTD triggers ER stress in human renal cancer cells.

### NCTD-induced ER stress leads to apoptosis of 786-O cells

**Figure 5:**
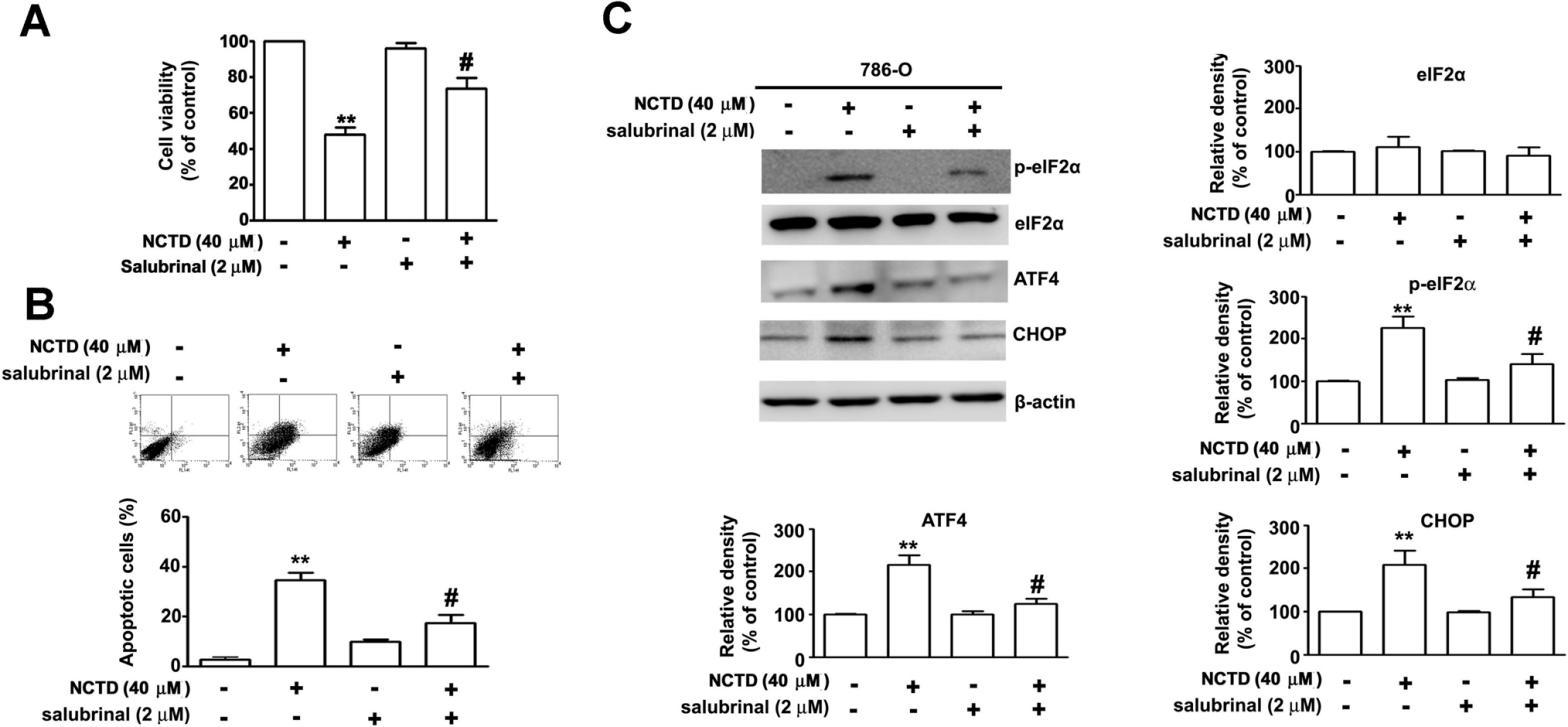
NCTD induces endoplasm reticulum stress-mediated apoptosis pathway 786-O cells were pretreated with salubrinal (2 μM) for 2 h before treated with NCTD (40 μM) for 24 h. (**A**) Cell viability was determined by MTT assay. (**B**) Apoptotic cells were detected by the Annexin-V and PI double-stained flow cytometry. (**C**) The level of ATF-4, CHOP, p-eIF2α and eIF2α expression were determined by western blot. β-actin used as an internal control. All data are represented as mean ± SEM (*n* = 3) for each group. ^**^p < 0.01 compared with control. ^#^*p* < 0.01 compared with NCTD.

Next, we sought to confirm that NCTD-induced ER stress leads to induction of apoptosis in renal cancer cells. Thus, we examined the effect of salubrinal, an ER stress inhibitor, on the response. The results show that salubrinal partly reversed the effect of NCTD on cell viability and apoptosis (Figure 5A). In agreement, annexin V/PI double staining indicated that salubrinal partly inhibited NCTD-induced apoptosis (Figure 5B), and western blotting showed that salubrinal partly reversed the effect of NCTD on expression of p-eIF2α, ATF-4, and CHOP in 786-O cells (Figure 5C). Overall, these results indicate that NCTD-induced ER stress is responsible for its induction of apoptosis.

### NCTD-induced AKT inactivation depends on ER stress-dependent apoptosis

**Figure 6:**
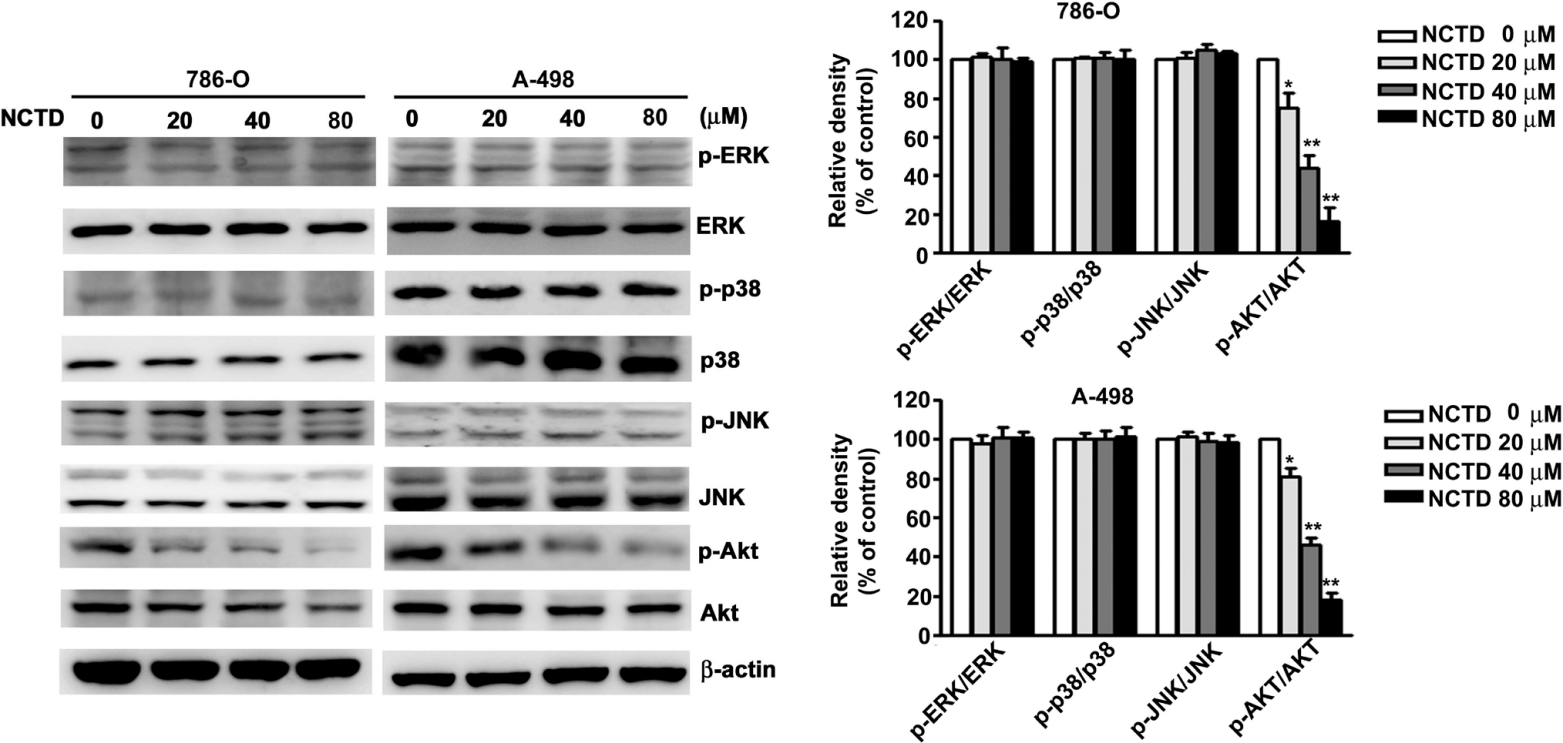
NCTD inhibit phosphorylation of AKT expression in 786-O and A-498 cells Cells were incubated with NCTD (0, 20, 40 and 80 μM) for 24 h and detected the total and phosphorylation of ERK, p38, JNK and AKT levels were measured by western blot in 786-O and A-498 cells. All data are represented as mean ± SEM (*n* = 3) for each group. ^**^p < 0.01 compared with control.

**Figure 7:**
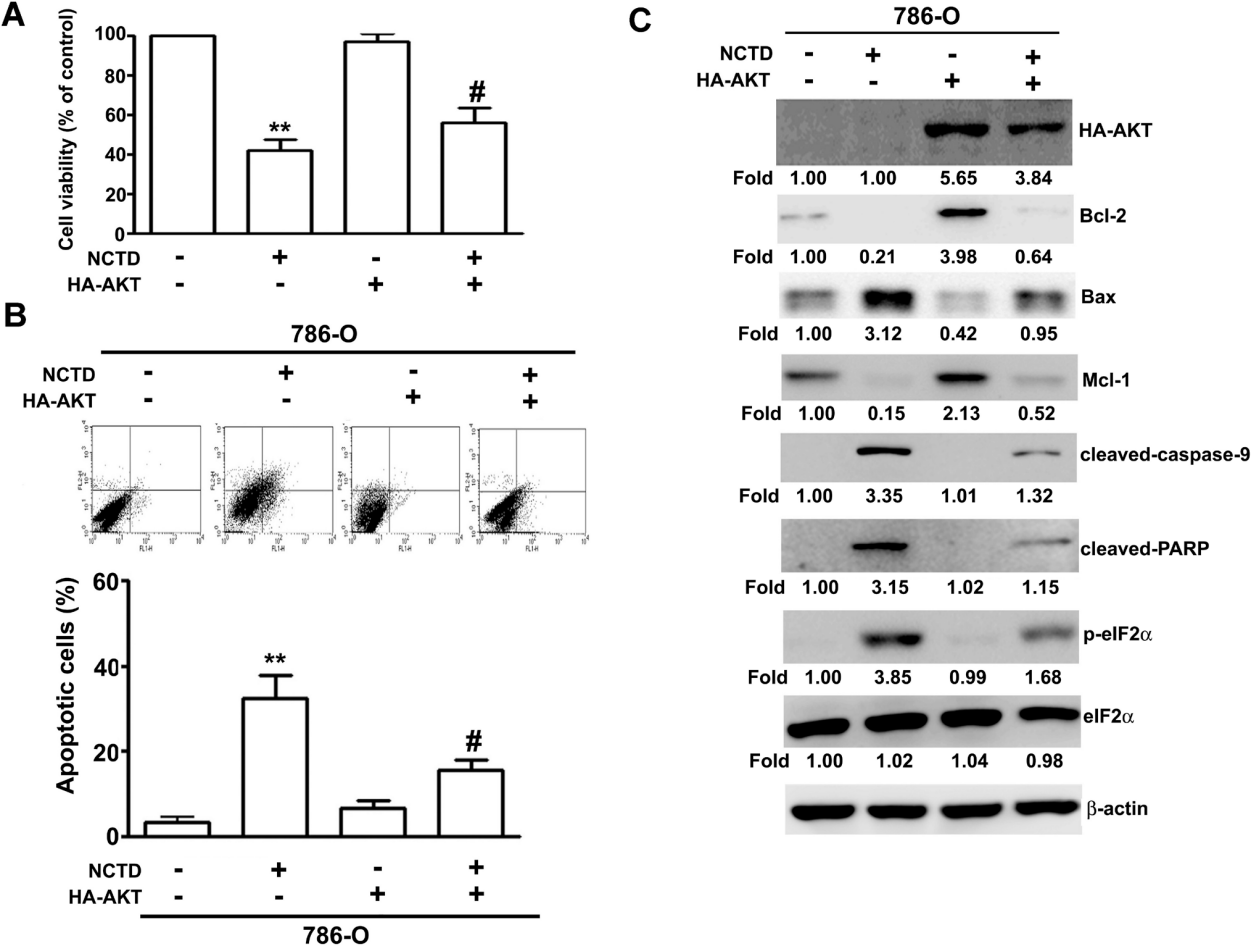
NCTD induces apoptosis through inactivation of AKT expression Transfection of Neo or HA-AKT plasmid in 786-O cells for 48 h, then followed by NCTD (40 μM) for another 24 h. (**A**) The cell viability was measured by MTT assay. (**B**) Apoptotic cells were detected by the Annexin-V and PI double-stained flow cytometry. (**C**) The expression of Bcl-2, Bax, Mcl-1, cleaved-caspase-9, cleaved-PARP, p-eIF2α and eIF2α were assessed by western blot. All data are represented as mean ± SEM (*n* = 3) for each group. ^**^p < 0.01 compared with control.

Next, we determined the role of the MAPK and AKT pathways on NCTD-induced apoptosis. Thus, we incubated 786-O and A-498 cells different concentrations of NCTD for 24 h, and performed western blotting analysis of proteins that have established roles in these pathways. The results show that NCTD reduced the activation of AKT in a dose-dependent manner, but did not affect ERK, p38, or JNK activation (Figure 6). We also investigated the role of ER stress on NCTD-inhibited AKT activation by simultaneous transfection of cells with a constitutive-AKT (HA-AKT) plasmid and treatment with NCTD (40 μM). The results show that the HA-AKT plasmid partly reversed the effect of NCTD on cell viability (Figure 7A) and apoptosis (Figure 7B). In addition, the HA-AKT plasmid also partly reversed the effect of NCTD on the expression of Bcl-2, Bax, p-eIF2α, cleaved-caspase-9, cleaved-PARP, and Mcl-1 (Figure 7C). These results demonstrate that AKT inactivation plays an essential role in NCTD-mediated apoptosis in human renal cancer cells.

### Antitumor effect of NCTD *in vivo*

**Figure 8:**
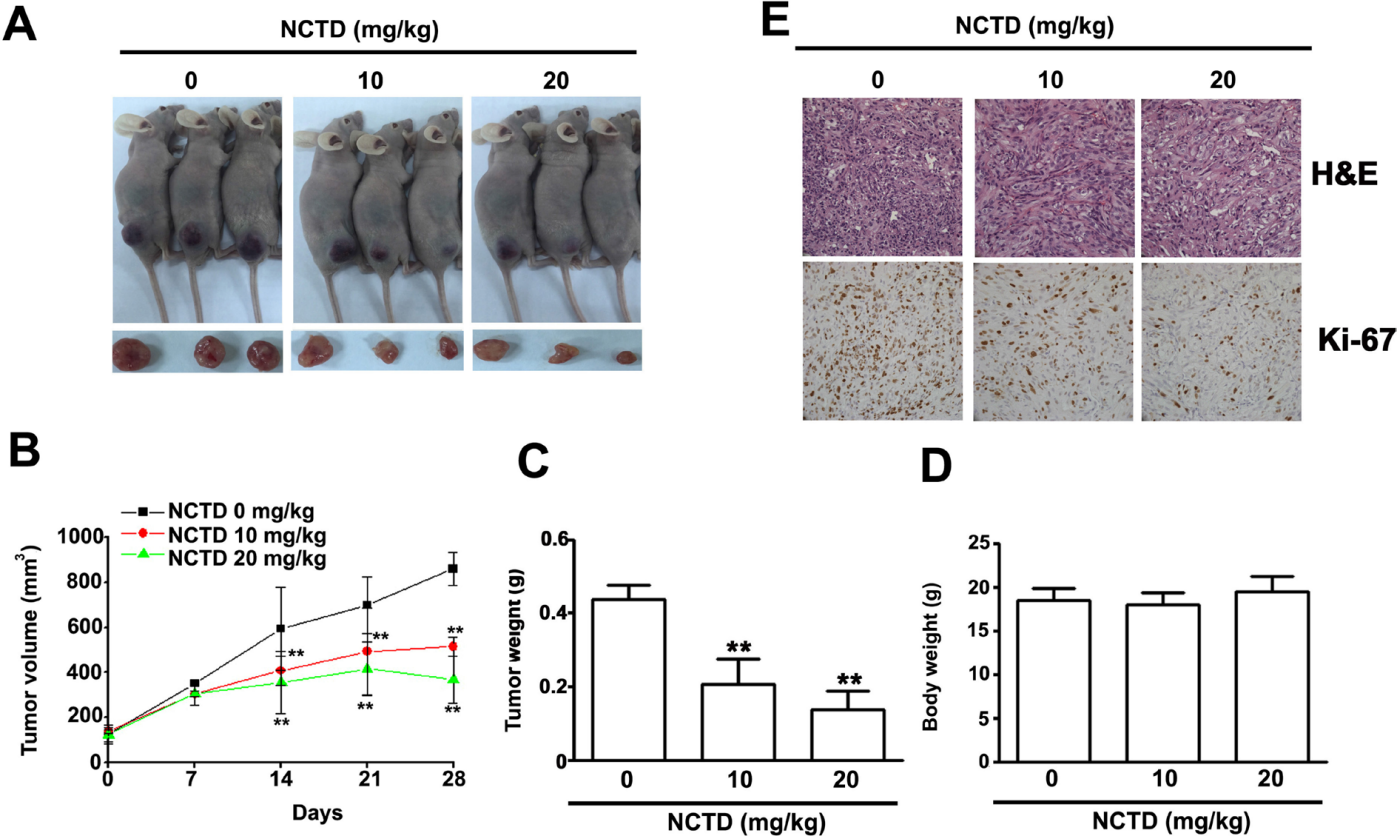
NCTD inhibits 786-O tumor xenograft growth *in vivo* (**A**) 786-O cells were injected to the right flanks of nude mice and palpable tumors formation were allowed to develop for 7 days, the nude mice bearing 786-O xenografts were orally NCTD (10 or 20 mg/kg) or DMSO twice times per week. (A) Visible tumor formation from mice at 28 days. (**B**) Tumor volumes and (**C**) Tumor weight in NCTD treated mice group were smaller than those of DMSO treated mice group. (**D**) Body weight of each group is shown. (**E**) H&E staining and immunohistochemical staining analysis on the expressions of Ki-67 and detect proliferation cells. Scale bars = 50 μm. ^**^p < 0.01, compared with DMSO groups.

Finally, we investigated the effect of NCTD on tumor growth *in vivo* using 786-O xenograft nude BALC/c mice (Figure 8A). After 28 days, tumor volume (Figure 8B) and weight (Figure 8C) were significantly lower in mice treated with NCTD at doses of 10 and 20 mg/kg, concentrations that did not significantly alter total body weight (Figure 8D). Immunohistochemistry indicated that Ki-67 was strongly inhibited at NCTD doses of 10 and 20 mg/kg (Figure 8E). These results indicate that NCTD reduced tumor growth *in vivo* at levels that had no apparent toxic effects.

## DISCUSSION

Additional anticancer drugs are needed to improve the outcome of patients with renal cancer. Many previous studies have reported that NCTD is a safe and effective treatment for many types of tumors [10], but little is known about its effect on renal cancer. Our results indicate that NCTD suppresses the growth of human renal cancer cells *in vitro* and *in vivo* by induction of cell cycle arrest and apoptosis, and this is due to its effect on ER stress and the AKT signaling pathway.

Mitochondria are important mediators of the intrinsic apoptosis pathway [25]. At the onset of apoptosis, there are changes in the outer mitochondrial membrane; in particular, changes in Bcl-2 family proteins alter the mitochondrial membrane potential [26]. This protein family has anti-apoptotic proteins, such as Bcl-xL and Bcl-2, and pro-apoptotic proteins, such as Bax [27]. Previous research on gastric cancer cells indicated that NCTD induced mitochondria-dependent cell apoptosis through activation of Bax and the release of cytochrome c, AIF, and Endo G into the cytosol [28]. In addition, Liu *et al*. found that NCTD increased apoptosis by alteration of TR3 and Bcl-2 in melanoma cells *in vitro* and *in vivo* [7]. Our experiments with 786-O cells indicate that NCTD treatment led to loss of the MMP, and that this was accompanied by an increase of the pro-apoptotic protein Bax, and decreases of the anti-apoptotic proteins Bcl-2 and Mcl-1.

The ER stress response occurs in diverse biological systems, and has roles in the regulation of cell proliferation, apoptosis, and autophagy in different types of tumor cells [23, 29]. Grp78 is a major regulator of ER stress. In particular, this protein functions as a molecular chaperone that maintains the integrity of the ER, and controls activation of UPR signaling molecules [30]. PERK dissociates from Grp78/BiP, and activates itself by oligomerization and phosphorylation, which directly phosphorylates the translation initiation factor eIF2α, leading to a general attenuation of protein synthesis [31]. However, activation of PERK also leads to increased expression of ATF4 and its targeted transcription factor CHOP (C/EBP homologous protein) during ER stress [32]. Our studies of 786-O cells demonstrated that NCTD-induced apoptosis correlated with ER stress and expression of ER stress-related proteins, such as p-eIF2α, CHOP, and ATF4 (Figure 4B). What is the relationship between NCTD-induced ER stress and apoptosis? NCTD induces the activation of Grp78 during the early stages of apoptosis, and then activates the transcription factor ATF4 and binding to the CHOP promoter. After this, there is an increase of mitochondrial membrane permeability due to dephosphorylation of AKT. Thus ATF-4/CHOP appears to mediate the apoptotic signals from the ER to the mitochondria. Another possibility is that anticancer drugs induce an excess of ROS, leading to increased apoptosis, possibly through activation of the ER stress-mediated apoptotic pathway [33, 34]. Inhibition of ER stress by salubrinal attenuated NCTD-induced apoptosis and the effect of NCTD on expression of eIF2a/ATF-4/CHOP. These results indicate that NCTD induced ER stress, and this led to increased apoptosis mediated by Grp78-phospho-eIF2α-ATF4-CHOP in 786-O cells.

PI3K/AKT activation plays an important role in the ER-stress-mediated regulation of different cell responses, such as proliferation, apoptosis, differentiation, and senescence [35, 36]. Recent studies found that inactivation of AKT is also required for the induction of ER stress-mediated apoptosis by certain phytochemicals, such as flavokawain C and cirsimaritin, and this is accompanied by inactivation of AKT [37, 38]. The phytochemical wogonin has potent cytotoxic effects, in that it induces ROS production and UPR activation through inhibition of AKT activation, leading to increased apoptosis of hepatocellular carcinoma cells [39] and HL-60 leukemia cells [40]. We found that NCTD inhibited the activation of AKT in 786-O and A-498 cells. Furthermore, overexpression of AKT attenuated the NCTD-induced apoptosis and ER stress in 786-O cells. These results suggest that NCTD induces apoptosis *via* the ER-dependent AKT apoptotic pathway.

In conclusion, our results demonstrate that NCTD-induced apoptosis in human RCC cells is mediated by induction of mitochondrial dysfunction, triggering of ER stress, and inactivation of AKT. These results provide important new insight into the possible molecular mechanisms of NCTD and highlight its potential use as an antitumor agent for RCC.

## MATERIALS AND METHODS

### Cell culture and reagents

The renal cancer cell 786-O (BCRC. 60243), A-498 (BCRC. 60241) and immortalized normal proximal tubule epithelial cells (HK-2, BCRC. 60097) were purchased from Bioresources Collection and Research Center (BCRC), Food Industry Research and Development Institute (Hsinchu, Taiwan). ACHN and CaKi-1 cells were kindly donated by Dr. M.H. Chien (Graduate Institute of Clinical Medicine, Taipei Medical University, Taipei, Taiwan). 786-O cells were maintained in RPMI-1640 (Gibco, Thermo Fisher Scientific, USA), and CaKi-1, A-498, and ACHN cells were maintained in MEM (Gibco, Thermo Fisher Scientific, USA) supplemented with 10% FBS (Hyclone, USA), 10 mM of glutamic acid, and 1% penicillin/streptomycin. All cells were maintained in a humidified atmosphere with 5% CO_2_ at 37°C.

NCTD, MTT, and JC-1 were purchased from Sigma (St. Louis, MO, USA). The pan-caspase inhibitor Z-VAD-FMK was purchased from BioVision (Mountain View, CA). Western blotting antibodies against p-p38, p-JNK, p-AKT, ERK1/2, p38, JNK1/2, AKT, Mcl-1, Bcl-2, Bax, and β-actin were purchased from Santa Cruz Biotechnology (California, USA). Antibodies against HA, p-ERK1/2, p-elF2α, elF2α, cleaved-caspase-3, -caspase-6, -caspase-7, -caspase-8, -caspase-9, and PARP were purchased from Cell Signaling Technology (Beverly, MA, USA).

### Cell viability assay

Cell growth was determined using the MTT assay, as previously described [41]. After treatment with NCTD for 24 h, 1 mL of the MTT reagent (0.5 mg/ml) was added to each well of a multi-well culture plate, and the plate was then incubated for 4 h at 37°C. The absorbance was determined at 570 nm using a Multiskan MS ELISA reader (Labsystems, Helsinki, Finland).

### Annexin-V/PI staining by flow cytometry

786-O and A-498 cells were seeded into 24-well plates and treated with different concentrations of NCTD (0, 20, 40, or 80 μM) for 24 h. Cells were then harvested and washed with PBS, and stained with FITC-labeled Annexin V/PI Apoptosis Detection kit (BD Biosciences, CA, USA) at dark light for 20 min. Samples were subjected to flow cytometry (FACSCalibur, BD, USA), and data were collected and analyzed on a BD FACSC using FACSD software.

### Measurement of Mitochondrial Membrane Potential

786-O and A-498 cells were treated with different concentrations of NCTD for 24 h. Cells were then harvested, washed, and incubated with 3 μg/mL of the JC-1 reagents. Samples were subjected to flow cytometry (FACSCalibur, BD, USA), and fluorescence was determined for FL1 (Green, FITC) at 530 nm and FL2 (Red, PE) at 590 nm, as well as CELLQuest software (FACSCalibur).

### Measurement of ER stress

After respective treatment cells were seeded on 8 well Lab-Tek Chambered coverglass (Thermo, Rochester, NY) and fixed with 4% paraformaldehyde, permeabilized with 0.1% Triton X100, stained with Hoechst 33342 reagent. The ER-ID Red Assay Kit (Enzo Life Sciences, Lörrach, Germany) was used for staining of the ER according to the manufacturer's instructions. Samples were mounted and photographed under an immunofluorescence microscopy. Quantify the fluorescence density of NCTD treated cells with ER-ID Red Assay Kit by FACSCalibur flow cytometer and the data were analyzed by Cell Quest software (BD Bioscience, Bedford, MA).

### Transfection assay

Cells seeded in 6-cm culture dish were transfected at 80% confluency with 3 μg HA-AKT plasmids using TurboFect transfection reagent (Thermo Fisher Scientific Inc.) according to the manufacturer's instructions. Twenty-four hours following transfection, cells were treated with NCTD (40 μM) and harvested after 24 h. AKT overexpression were monitored and verified by western blotting analysis.

### Western blot analysis

Western blotting analyses were performed as described previously [42]. Equal amounts of protein extracts (25 μg) were subjected to 10% or 12% SDS-PAGE, and then blotted onto PVDF membranes. The membranes were blocked for 1 h at room temperature using 5% nonfat milk in PBST, and then incubated with the primary antibodies in TBST overnight at 4°C. Proteins were then detected by enhanced chemiluminescence using the Immobilon Western-HRP Substrate (Millipore, Billerica, MA, USA).

### Xenograft mouse model

A xenograft mouse model was used to evaluate the effect of NCTD on tumor growth *in vivo*, as previous described [15]. Each BALB/c male mouse received a subcutaneous inoculation of 786-O cells (5 × 10^6^ /0.1 mL) for 7 days. Mice were divided into three groups, with five animals per group. These mice received 0, 10, or 20 mg/kg body weight of NCTD by oral gavage twice per week. When the tumors of control group reached around 800 mm^3^, all mice were killed and the tumors were excised and weighed.

### Immunohistochemistry

Immunohistochemical studies were performed as previously described [43]. Tissues were fixed in 4% paraformaldehyde, embedded in paraffin, and sectioned at a thickness of 4 μm. These sections were then deparaffinized, hydrated, and immersed three times in PBS. Then, the antigen was retrieved by pre-treatment in a microwave oven for 10 min with 10 mM citrate buffer (pH 6.0). The sections were incubated in 3% H_2_O_2_ for 10 min at room temperature to eliminate the activity of endogenous peroxidase. After washing in PBS, the sections were blocked in 5% normal goat serum for 15 min at room temperature. Slides were drained and incubated with the primary antibody anti-Ki-67 (1:200) at 4°C overnight. Then, the slides were incubated in the secondary antibody at 37°C for 15 min, and peroxidase activity was visualized by adding a standard diaminobenzidine/hydrogen peroxide solution for 2 min. The sections were counterstained with hematoxylin, and observed under a light microscope (Nikon, Japan).

### Statistical analysis

All experiments were repeated at least three times independently. Data are expressed as means ± standard deviations and analyzed with SPSS version 10.0 (SPSS, Inc., Chicago, IL, USA) and GraphPad PRISM (version 6.0; Graph Pad Software) by Student's *t*-test or analysis of variance (ANOVA). A *p* value of 0.05 or 0.01 was considered significant.
